# Visualization and Cluster Analysis of Peroxisome Proliferator–Activated Receptors in Colorectal Cancer: Research Trends and Future Directions

**DOI:** 10.1155/humu/9971217

**Published:** 2026-04-24

**Authors:** Bo Dong, Bin Zhou, Hong Zhang, Taiqi Wang, Zhaowan Xu, Lin Zhu, Qingyun Li, Ben Niu, Xiaofeng Sun

**Affiliations:** ^1^ Department of Gastro-Pancreatic Surgery, Shanxi Provincial People′s Hospital Affiliated to Shanxi Medical University, Taiyuan, Shanxi Province, China; ^2^ Laboratory of Digestive Surgery, State Key Laboratory of Biotherapy and Cancer Center, West China Hospital, West China School of Medicine, Sichuan University, Chengdu, Sichuan Province, China, scu.edu.cn; ^3^ School of Medicine, Institute of Medical Sciences, Örebro University, Örebro, Sweden, oru.se; ^4^ Department of Oncology and Department of Biomedical and Clinical Sciences, Linköping University, Linköping, Sweden, liu.se

**Keywords:** bibliometric analysis, CiteSpace, colorectal cancer, peroxisome proliferator–activated receptors, VOSviewer

## Abstract

**Objective:**

Peroxisome proliferator–activated receptors (PPARs) are essential regulators in the development and progression of colorectal cancer (CRC). However, a comprehensive bibliometric analysis of PPAR‐related CRC research is lacking.

**Methods:**

Publications on PPARs in CRC from 1998 to 2024 were retrieved from the Web of Science Core Collection. VOSviewer, CiteSpace, and the R package bibliometrix were used to analyze publication trends, hotspots, and collaboration networks. Contributions from countries, institutions, authors, and journals were systematically evaluated.

**Results:**

A total of 1380 publications were analyzed, with an annual growth rate of 8.33%. The United States led with the highest number of publications (386) and citations (28,461), followed by China and Japan. The University of Texas System was the most productive institution (125 publications). Gonzalez, Frank J. and Peters, Jeffrey M. were the most prolific authors (16 publications each). *Cancer Research* was the leading journal. Cluster analysis of keywords identified three major themes: regulatory mechanisms of PPARs in CRC, the association with obesity, and therapeutic applications. Current research hotspots included proliferation, inflammation, oxidative stress, and ulcerative colitis.

**Conclusion:**

This study highlights three key research focuses: molecular mechanisms of PPARs in CRC, the link between obesity and CRC, and therapeutic potential. These findings underscore the growing interest in PPAR‐mediated metabolic regulation, inflammation, and targeted interventions, offering valuable guidance for future research directions.

## 1. Introduction

Colorectal cancer (CRC) is recognized as one of the most prevalent and fatal malignancies globally, ranking as the third most common cancer and the second leading cause of cancer‐related mortality worldwide [1, 2]. The pathogenesis of CRC is characterized by intricate interactions among genetic predisposition, environmental factors, and lifestyle choices, which influence multiple biological pathways [3]. Despite advancements in screening, diagnosis, and treatment, current therapeutic approaches for CRC face significant limitations. Conventional chemotherapeutic agents like 5‐fluorouracil and oxaliplatin are associated with severe toxicity and acquired resistance, whereas targeted therapies such as anti‐EGFR (cetuximab and panitumumab) and anti‐VEGF (bevacizumab) agents benefit only specific patient subsets and often develop resistance mechanisms [4]. Furthermore, immune checkpoint inhibitors show efficacy primarily in microsatellite instability–high tumors, representing merely 15% of CRC cases [5]. These therapeutic constraints, combined with rising CRC incidence and mortality rates, underscore the urgent need for identifying novel molecular targets that can overcome existing limitations and provide broader therapeutic benefits.

Peroxisome proliferator–activated receptors (PPARs) are ligand‐activated nuclear receptors that are integral to the regulation of cellular metabolism, inflammation, proliferation, and differentiation [6–8]. The PPAR family comprises three subtypes: PPAR*α*, PPAR*γ*, and PPAR*δ*/*β*, each exhibiting distinct tissue distribution patterns and physiological functions [9]. The relationship between PPARs and CRC has attracted considerable scholarly attention in recent decades. PPARs have been demonstrated to influence various facets of CRC pathogenesis, encompassing cell proliferation, apoptosis, angiogenesis, and inflammation [10]. For instance, the activation of PPAR*γ* has exhibited antitumorigenic effects in CRC through multiple mechanisms, such as inducing cell cycle arrest and facilitating cell differentiation [11]. In contrast, PPAR*δ*/*β* has been shown to exert context‐dependent effects, with the potential to either promote or suppress CRC development based on specific molecular and cellular environments [12]. The therapeutic potential of PPAR modulators in CRC treatment has emerged as a significant area of research interest. Numerous PPAR agonists have been explored as potential therapeutic agents, both as monotherapy and in combination with conventional treatment modalities [13]. However, the intricate roles of PPARs in CRC and their interactions with diverse signaling pathways necessitate a thorough understanding of the current research landscape.

Bibliometric analysis represents a powerful tool for evaluating scientific literature through quantitative analysis, helping researchers identify crucial contributors, collaborative networks, and emerging research trends [14]. Although several reviews have examined the role of PPARs in CRC research [15, 16], they often lack data‐driven visualizations and rely heavily on subjective interpretation. This limits the objectivity and comprehensiveness of their findings. To overcome these shortcomings, this study employs bibliometric analysis to provide a comprehensive overview of PPARs research in CRC, mapping key contributors, research output, emerging hotspots, and frontiers of the field.

## 2. Material and Methods

### 2.1. Data Source and Search Strategy

The literature search was performed on the Web of Science Core Collection (WoSCC) website to identify publications indexed between January 1, 1998, and September 24, 2024. The specific search formula was as follows: Topic (TS) = ((“peroxisome proliferator‐activated receptor∗” OR PPAR)) OR NR1C1 OR NR1C2 OR NR1C3)) AND TS = ((Rectal∗ OR Rectum∗ OR Colorectal∗ OR Colonic∗ OR Colon∗ OR colo‐rectal∗) AND (Neoplas∗ OR Tumo∗ OR Cancer∗ OR Carcinoma∗ OR adenoma∗ OR adenocarcinoma)) [17–19]. The literature retrieval was performed on a single day (September 24, 2024) to minimize variations resulting from database updates. This analysis only included original research articles on PPARs in CRC indexed in the WoSCC database. Review, letters, commentaries, editorials, conference abstracts, and studies published under similar or different titles in various journals were excluded.

### 2.2. Data Extraction and Bibliometric Analysis

The extracted bibliometric parameters included journal names, publication times, titles, countries/regions, institutions, authors, keywords, references, and citations. Journal impact factors (IFs) were collected from the most recent Journal Citation Reports (2023). Microsoft Excel 2019, VOSviewer (Version 1.6.20), CiteSpace (Version 6.3 R1), and R bibliometrix were used to perform the bibliometric analysis and visualization. The R bibliometrix package was employed to analyze country‐specific metrics, including the number of publications, citations, and international collaborations [20]. For the VOSviewer analysis, we set the minimum number of documents per author to 2 and the minimum number of citations per author to 20. For keyword analysis, we set the minimum occurrence to five. The network visualization was based on bibliographic coupling, where the relatedness of items was determined by the number of shared references. The clustering of research themes was performed using VOSviewer′s clustering algorithm with a resolution parameter of 1.0. The average publication year of keywords was calculated to track the evolution of research topics over time. The trending topics were identified by analyzing keywords with strong citation bursts in recent years (2018–2024). CiteSpace was used to visualize keyword and reference burst figures and reference cocitation networks. The following parameters were set in CiteSpace: time slicing (1998–2024), years per slice (1), term source (author keywords and Keywords Plus), node type (cited reference), selection criteria (g − index with k = 25), and pruning (pathfinder and pruning the merged network) [21]. For the analysis of research hotspots and trends, we used the “Keywords Plus” provided by WoSCC rather than author keywords, as it provided more comprehensive coverage of research topics. The burst detection algorithm in CiteSpace was applied to identify emerging trends and sudden increases in the frequency of specific topics. The H‐index was utilized to quantify the academic impact of both individuals and journals. The H‐index serves as a crucial metric for assessing the scholarly contributions of researchers and has the potential to predict their future scientific accomplishments [22, 23].

## 3. Results

### 3.1. Overall Characteristics

**Figure 1:**
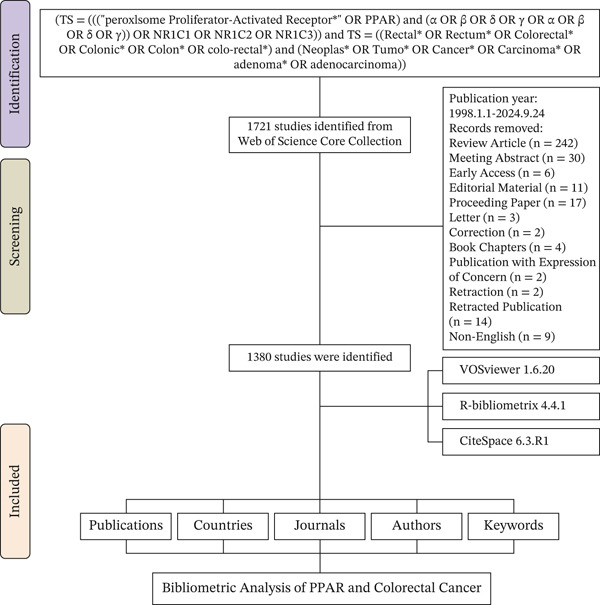
Flowchart of the literature screening process.

Of the 1721 publications initially identified, 341 were excluded, and 1380 were finally included in the analysis (Figure 1). The investigation showed that 8486 authors from 4321 institutions across 332 countries/regions contributed to the production of these manuscripts. These works were published in 467 journals, citing 43,059 references (Figure 2a). The number of published articles showed an overall upward trend, with the peak of 81 publications occurring in 2006 (Figure 2b).

Figure 2Analysis of general information. (a) Summary information of the included studies. (b) Annual number of publications.

**Figure:**
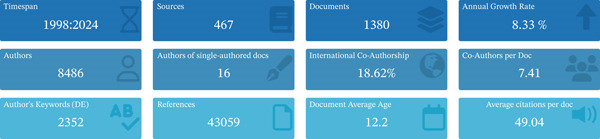
(a)

**Figure:**
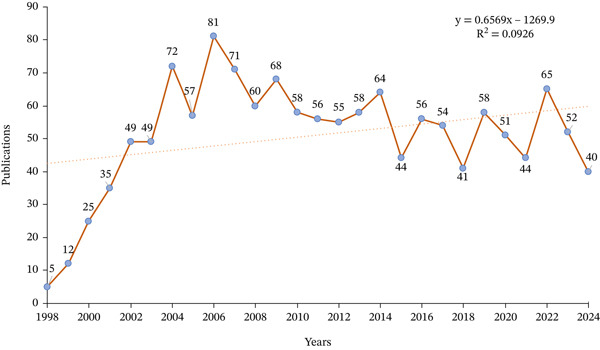
(b)

### 3.2. Countries/Regions Analysis

In this study, 332 countries/regions have contributed to this field. The United States dominated with 386 publications (28.0%), followed by China (306, 22.2%) and Japan (161, 11.7%) (Figure 3a). Regarding total citations, the United States ranked first (28,461), followed by China (7323) and Japan (8181). For average citations per paper, Belgium led with 127.2, followed by the United States (73.7) and France (73.1) (Table S1). Among the 54 countries involved in international collaborations with a minimum of one article, the United States had the highest number of collaborations with other countries (link strength[ ] = 217), followed by China (LS = 81) and Germany (LS = 64) (Figure 3b). Table S2 presents the country clusters, and Figure S1 presents the distribution of the high‐frequency keywords among the clusters. Specific keyword patterns can be seen among the country clusters. For example, Cluster 10 (the United States, Mexico, and Argentina) was characterized by frequent use of keywords such as “activated receptor gamma,” “gene expression,” “expression,” “differentiation,” and “in vitro,” whereas Cluster 8 (China, Ireland, and New Zealand) showed a thematic emphasis on functional cellular outcomes, evidenced by the prominence of keywords such as “growth,” “proliferation,” “apoptosis,” and “cells.”

Figure 3Analysis of countries. (a) Distribution of corresponding author′s publications by country. (b) Visualization map depicting the collaboration among different countries.

**Figure:**
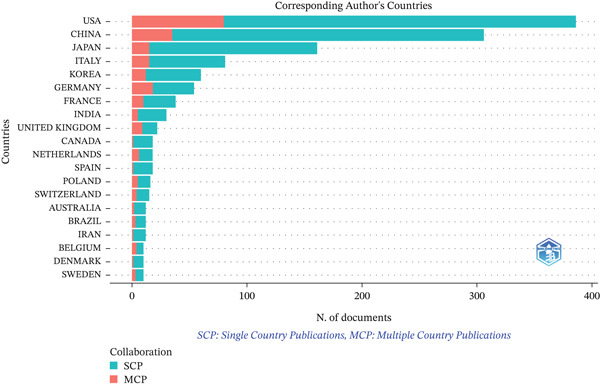
(a)

**Figure:**
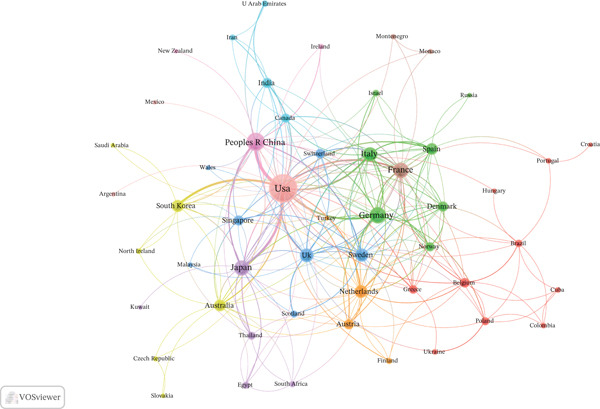
(b)

### 3.3. Institutional Analysis

Among the 4321 participating institutions, the University of Texas System contributed the most publications (LS = 125), followed by Harvard University (LS = 115) and UTMD Anderson Cancer Center (LS = 96) (Figure 4a). Among the 130 institutions involved in international collaborations with a minimum of five articles, Harvard University had the highest number of collaborations with other countries (LS = 55), followed by the National Cancer Institute (NCI) (LS = 44) and Brigham and Women′s Hospital (LS = 32) (Figure 4b).

Figure 4Analysis of institutions. (a) Top 10 institutions by article count and rank. (b) Visualization map depicting the collaboration among different institutions.

**Figure:**
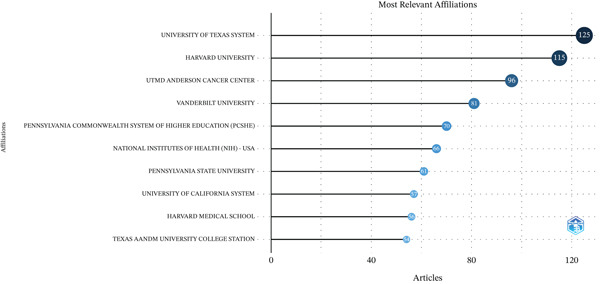
(a)

**Figure:**
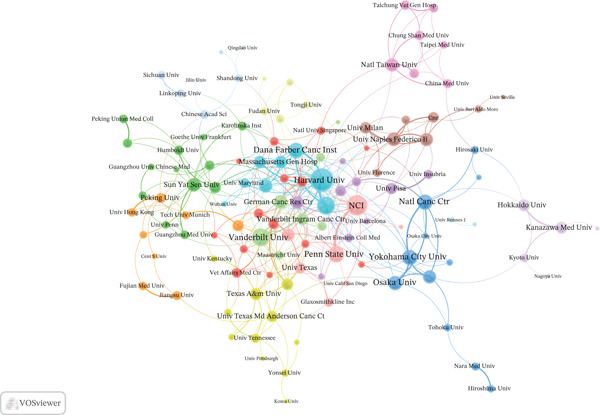
(b)

### 3.4. Journal Analysis

A total of 467 journals published articles in this field. *Cancer Research* led with 45 publications, followed by the *Journal of Biological Chemistry* (33) and *Carcinogenesis* (29). Regarding IFs and citations, *Cancer Research* (IF = 12.5, 2973 citations) maintained its leading position, whereas the *Journal of Biological Chemistry* (IF = 4, 3165 citations) and *Clinical Cancer Research* (IF = 10, 677 citations) also showed strong performance (Table S3). The co‐occurrence networks of journals contain 125 with at least three occurrences. The three principal journals exhibiting the highest total LS within the co‐occurrence networks were *Cancer Research* (646), *Nature Medicine* (603), and *Journal of Biological Chemistry* (367) (Figure 5a). The coupling networks of journals contain 125 with at least three couples. The three principal journals with the highest total LS were *Cancer Research* (31,348), *Journal of Biological Chemistry* (24,859), and *International Journal of Cancer* (16,196) (Figure 5b).

Figure 5Analysis of journals. (a) Co‐occurrence network of journals. (b) Coupling network of journals.

**Figure:**
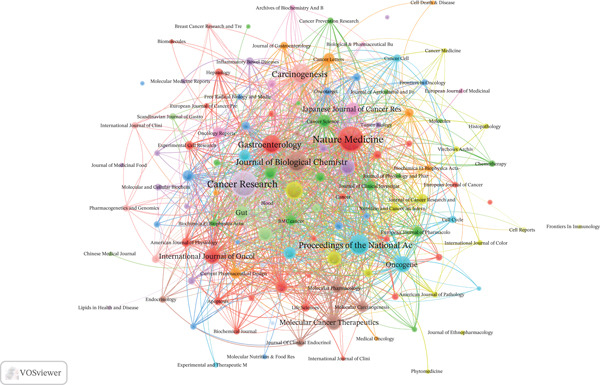
(a)

**Figure:**
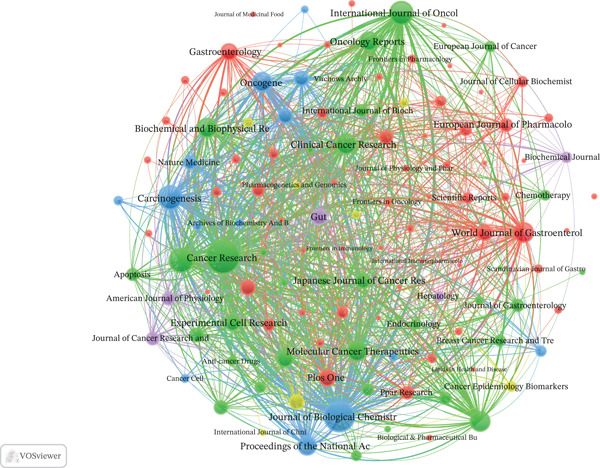
(b)

### 3.5. Authors Analysis

**Figure 6:**
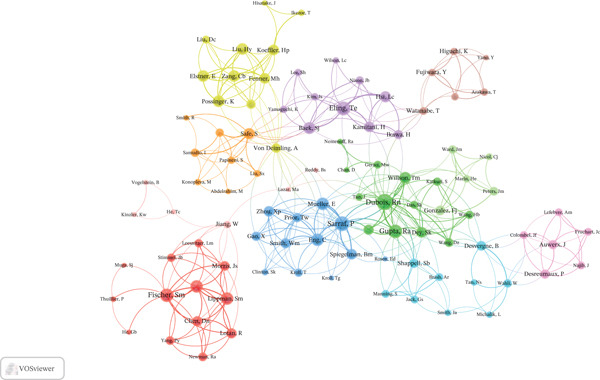
Visualization map depicting the collaboration among different authors.

The publication and citation profiles of high‐impact authors were presented in Table S4. Gonzalez, Frank J. and Peters, Jeffrey M. emerged as the most prolific authors, each contributing 16 publications, whereas Sarraf P had the highest citation impact (2816 citations). The collaboration network analysis revealed that among the 1012 authors involved in international collaborations with a minimum of two articles, Gonzalez, Frank J. had the highest number of collaborations with other authors (LS = 83), followed by Colantuoni, Vittorio (LS = 68) and Peters, Jeffrey M. (LS = 62) (Figure 6).

### 3.6. Keyword Analysis

The keyword co‐occurrence analysis identified 131 keywords appearing at least 15 times, clustering into three main research themes (Figure 7a). Cluster #1 represented PPAR regulatory mechanisms, including “inhibition,” “expression,” and “downregulation.” Cluster #2 focused on the relationship between metabolic diseases, particularly obesity, and cancer, emphasizing how obesity and its associated metabolic abnormalities influence cancer initiation and progression. The primary keywords included “Obesity,” “Insulin Resistance,” and “Adipokines.” Cluster #3 centered on the key role of PPARs in cancer, as well as on drug therapies and other cancer‐related mechanisms. Its characteristic keywords included “Apoptosis,” “NF‐kappa B,” “Human Breast Cancer,” and “Troglitazone.”

Figure 7Analysis of keywords. (a) Visual analysis of keyword co‐occurrence network analysis. (b) Top 20 keywords with the strongest citation bursts.

**Figure:**
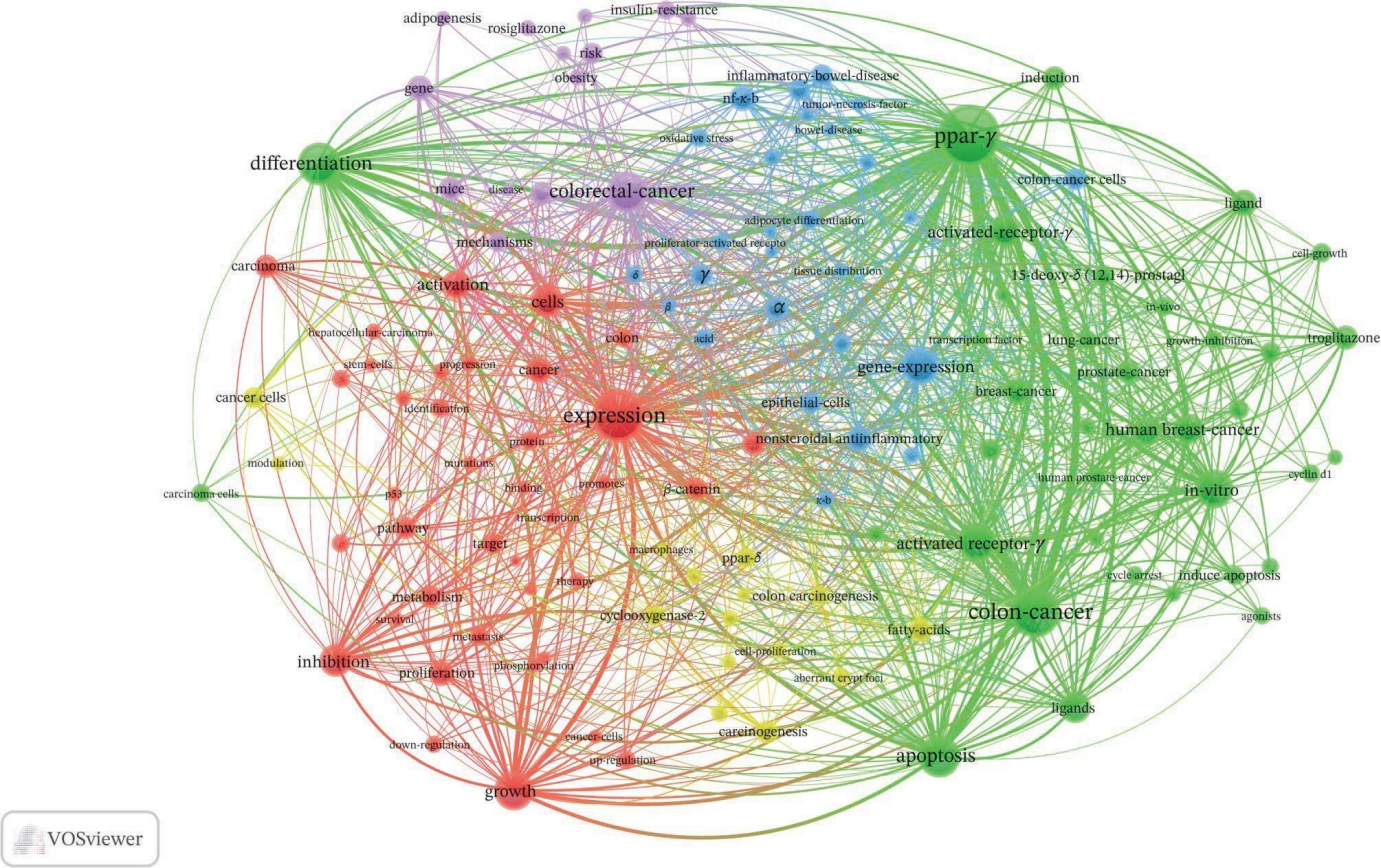
(a)

**Figure:**
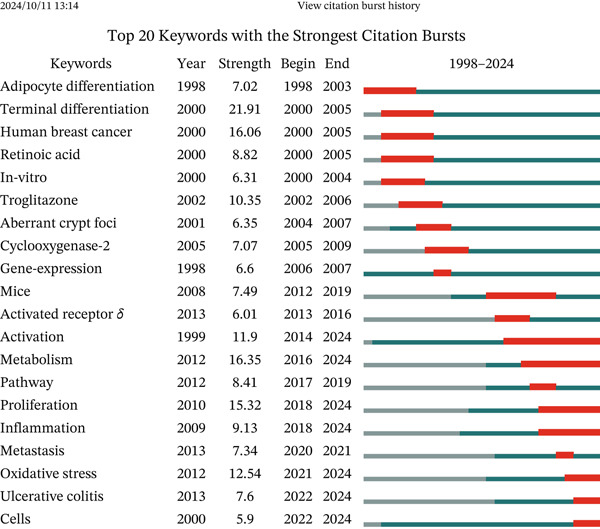
(b)

The burst analysis of keywords revealed an evolution from fundamental molecular mechanisms (1998–2003) to therapeutic applications (2004–2013) and subsequently to precision medicine approaches (2014–2024). Early studies primarily concentrated on topics such as “adipocyte differentiation,” “terminal differentiation,” and “human breast cancer.” However, in recent years, there has been a gradual shift in focus toward areas associated with disease metabolism and inflammation, encompassing “metastasis,” “oxidative stress,” “ulcerative colitis,” and “cells.” Notably, the periods of “activation,” “metabolism,” and “inflammation” extend over an extended timeframe, underscoring their continued relevance in biomedical research (Figure 7b).

## 4. Discussion

This bibliometric analysis indicates a consistent increase in the volume of publications, underscoring the growing acknowledgment of the significance of PPARs in CRC biology. The United States demonstrates a leading position in terms of publication output and citation impact, followed by China and Japan, which highlights global disparities in research infrastructure and funding. Robust collaborative networks, particularly among major institutions such as the University of Texas System and Harvard University, have been instrumental in enhancing the understanding of PPAR signaling and its implications in the development of CRC [24, 25]. The keyword clustering analysis revealed three distinct research themes that define the current landscape of PPAR research in CRC: molecular mechanisms of PPAR regulation, including inhibition and expression pathways; the critical relationship between obesity‐related metabolic dysfunction and cancer development through PPAR signaling; and the therapeutic potential of PPAR modulators in cancer treatment and prognosis. These findings indicate that future research should prioritize the elucidation of precise molecular mechanisms underlying PPAR‐mediated colorectal carcinogenesis, the development of personalized therapeutic strategies based on metabolic profiling, and the optimization of PPAR‐targeted interventions to overcome current treatment limitations and improve patient outcomes.

### 4.1. Cluster 1: Molecular Mechanism of PPARs in CRC

The prominence of keywords such as “inhibition,” “expression,” and “downregulation” indicated a significant scholarly interest in investigating the molecular mechanisms through which PPARs regulate CRC. With advances in molecular biology, researchers have elucidated the complex roles of PPARs in the development and progression of CRC. Numerous signaling cascades have been identified as playing critical roles in this process. Cheng et al. demonstrated that PPARs function as pivotal regulators within the tumor microenvironment, mediating both metabolic reprogramming and inflammatory responses [26]. The significance of the tumor microenvironment has increasingly garnered attention, with studies indicating that PPAR‐mediated signaling molecules can facilitate cancer progression through autocrine or paracrine pathways. Recent studies have elucidated several critical molecular mechanisms. For instance, Chi et al. demonstrated that activation of PPAR*γ* can effectively inhibit cancer cell proliferation and induce apoptosis via multiple downstream pathways [11]. Furthermore, Wagner N. and Wagner K‐D established that PPARs act as essential regulators in the pathological progression of CRC, particularly through their modulation of inflammatory pathways [27].

### 4.2. Cluster 2: The Relationship Between PPAR, Obesity, and CRC

PPARs serve as a crucial link between obesity and CRC, functioning as a key regulator in the onset and progression of both conditions [28]. A study by Motawi et al. revealed that the downregulation of PPAR*γ* is closely associated with an increased susceptibility to CRC in obese individuals [29]. Furthermore, single‐cell sequencing of primary and metastatic CRC tumors conducted by Wang et al. demonstrated significant upregulation of the PPAR signaling pathway‐related genes in tumor epithelial cells [30]. These findings suggest that PPARs may play a dual role in the development and progression of obesity‐related CRC, potentially offering protective effects under certain conditions while promoting carcinogenesis in others. Clinically, PPAR agonists have garnered attention as potential therapeutic targets for obesity and cancer, highlighting their significant research value. However, further investigation is required to elucidate the balance between their anticancer effects and metabolic regulatory functions to develop safe and effective treatment strategies.

### 4.3. Cluster 3: The Potential of PPAR in Cancer Prognosis and Treatment

PPARs are integral to the initiation and advancement of CRC, breast cancer, and prostate cancer, thereby positioning themselves as significant prognostic biomarkers and therapeutic targets [31]. The incorporation of PPAR regulators, such as rosiglitazone, alongside conventional therapies, especially those designed to modulate PPAR‐mediated inflammatory pathways, has yielded substantial therapeutic benefits [10]. Future treatment strategies are expected to concentrate on inhibiting the progression of CRC through the modulation of PPARs, with particular emphasis on enhancing apoptotic processes. As elucidated by Zhang et al., long noncoding RNA TINCR modulates PPAR signaling via the miR‐107/CD36 axis, thereby contributing to the suppression of CRC progression [32]. Furthermore, the interaction between PPARs and nuclear factor kappa‐light‐chain‐enhancer of activated B cells (NF‐kappa B) is expected to become a pivotal area for future research. Current investigations are focused on the development of more specific and effective PPAR modulators while integrating immunotherapy and targeted therapies, with the aim of reducing side effects and further improving treatment efficacy [13].

Distinct thematic orientations were evident across international collaboration clusters. Cluster 10, comprising the United States, Mexico, and Argentina, was characterized by frequent use of keywords such as “activated receptor gamma,” “gene expression,” “expression,” “differentiation,” and “in vitro” [33–38]. This pattern indicates a research focus on the mechanistic roles of PPAR*γ* activation in colorectal cancer, emphasizing transcriptional regulation and cellular differentiation processes predominantly investigated through in vitro experimental models. The strong presence of terms related to receptor activation and gene expression reflects a mechanistic, hypothesis‐driven research paradigm, aligning with these countries′ established strengths in molecular and nuclear receptor biology. In contrast, Cluster 8, involving China, Ireland, and New Zealand, showed a thematic emphasis on functional cellular outcomes, evidenced by the prominence of keywords such as “growth,” “proliferation,” “apoptosis,” and “cells” [39–43]. Rather than concentrating on upstream receptor activation, this collaboration cluster appears to focus on downstream phenotypic consequences of PPAR‐related signaling, particularly those affecting tumor cell growth and survival. This orientation suggests a more application‐ and outcome‐driven research strategy, reflecting translational interests in tumor progression and therapeutic response. Collectively, these contrasting patterns reveal that international collaboration clusters in PPAR‐related CRC research specialize along a continuum, from receptor‐centered mechanistic studies to phenotype‐focused functional investigations. Such thematic complementarity underscores the scientific value of cross‐cluster collaboration in linking molecular mechanisms to biologically and clinically relevant outcomes.

The present bibliometric analysis delineates three principal, evidence‐based directions for future research on PPARs in CRC: (1) isoform‐ and genotype‐specific modulation of PPARs (particularly PPAR*δ* variants) through next‐generation small‐molecule agents or targeted degraders; (2) integration of PPAR‐mediated metabolic reprogramming with immunotherapeutic and tumor‐microenvironmental strategies; and (3) development of biomarker‐driven, multiomic precision approaches linking PPAR signaling to prognosis, therapeutic response, and resistance mechanisms [44, 45]. The temporal evolution of research topics indicates a distinct transition from early studies focused on “PPAR*γ* and colon carcinogenesis” to more recent emphases on “metabolic reprogramming,” “immune microenvironment,” “single‐cell sequencing,” and “immunotherapy,” reflecting a shift from descriptive molecular biology toward therapeutic and precision–oncology applications. Co‐occurrence and clustering of terms such as “PPAR agonists,” “lipid metabolism,” “NF‐*κ*B,” and “PD‐1/PD‐L1” suggest that future investigations will increasingly explore combination regimens targeting both PPAR‐regulated metabolic pathways and mechanisms of immune evasion, rather than relying on monotherapy with classical thiazolidinediones [44, 46, 47]. Accumulating evidence underscores the divergent biological functions of PPAR isoforms, with PPAR*γ* activation generally exerting tumor‐suppressive effects, whereas PPAR*β*/*δ* signaling may enhance proliferation, stemness, and metastatic potential under specific molecular or genetic contexts. The identification of functional polymorphisms such as PPAR*δ*‐87T/C, which modify CRC susceptibility and transcriptional output, supports the rationale for genotype‐stratified studies examining PPAR modulators in conjunction with dietary, microbiomic, and standard therapeutic factors. The emerging prominence of keywords including “ligand design,” “PROTAC,” and “selective modulators” further highlights a methodological shift toward mutation‐ and isoform‐selective pharmacologic targeting. Priority areas for future investigation include the development of PPAR*δ*‐selective antagonists or degraders tested in variant‐defined populations, multiomic dissection of resistance mechanisms to PPAR*γ* agonists, and comparative analyses of pan‐PPAR versus isoform‐specific modulation in organoid and in vivo CRC models [44–46]. Finally, the increasing appearance of terms such as “biomarker,” “prognosis,” and “risk model” aligns with the expanding effort to incorporate PPAR‐related molecular features into integrated predictive tools. Such frameworks, combining isoform expression, pathway activity signatures, and genomic–metabolomic correlates, may refine patient stratification and therapeutic decision‐making in CRC, thereby advancing a precision–medicine paradigm for PPAR‐directed interventions.

This bibliometric analysis demonstrates several key strengths that enhance its validity and impact. First, the study employed rigorous methodology utilizing the comprehensive WoSCC database and multiple advanced analytical tools (VOSviewer, CiteSpace, and R bibliometrix) for cross‐validation and triangulation of results. Second, the extensive 26‐year timespan (1998–2024) captured the complete evolutionary trajectory of PPAR research in CRC, providing valuable temporal insights into emerging trends and research hotspots. Finally, the large sample size of 1380 publications from 8486 authors across 332 countries ensures statistical robustness and global representativeness of findings.

However, several limitations of our study should be acknowledged. First, our analysis was restricted to WoSCC database publications, potentially missing relevant papers indexed in other databases such as PubMed, Scopus, or Embase, which may contain unique content not covered by WoSCC. Second, the focus on English‐language publications may have excluded valuable contributions in other languages, particularly research from non‐English–speaking countries that could provide different perspectives on PPAR research in CRC. Third, citation metrics might not fully capture a publication′s clinical or practical impact, as highly cited papers may not necessarily translate to clinical utility or therapeutic breakthroughs. Fourth, the bibliometric analysis cannot assess the quality or validity of individual studies, potentially including flawed research alongside high‐quality investigations. Fifth, the keyword analysis relies on author‐selected terms and database indexing, which may not always accurately represent the actual research content or may be influenced by publication trends and keyword popularity. Finally, the focus on article publications excluded other important scientific communications such as conference presentations, patents, and clinical trial reports that may contribute significantly to the field’s advancement.

## 5. Conclusion

This bibliometric analysis mapped global research on PPARs in colorectal cancer, identifying 1380 publications from 8486 authors across 332 countries. The United States, China, and Japan led the field, with major contributions from institutions such as the University of Texas System, Harvard University, and MD Anderson Cancer Center, and key journals including Cancer Research and the Journal of Biological Chemistry. Three main research themes emerged: (1) molecular mechanisms of PPAR regulation in CRC, including signaling pathways and metabolic reprogramming; (2) links between obesity, metabolic dysfunction, and CRC via PPAR signaling; and (3) therapeutic potential of PPAR modulators and prognostic biomarkers. Over time, the field has evolved from basic molecular studies to mechanistic and translational research emphasizing metabolism, inflammation, and precision oncology. Overall, this shift toward deeper a mechanistic understanding reflects the growing consensus that elucidating PPAR‐mediated pathways is vital to advancing targeted therapies and improving CRC outcomes.

## Author Contributions

Bo Dong and Bin Zhou carried out the studies, participated in collecting data, and drafted the manuscript. Hong Zhang, Taiqi Wang, Zhaowan Xu, Lin Zhu, Qingyun Li, and Ben Niu performed the statistical analysis and participated in its design. Bo Dong and Xiaofeng Sun participated in the acquisition, analysis, or interpretation of data and drafted the manuscript. Bo Dong and Bin Zhou contributed equally to this work.

## Funding

This study was supported by the Provincial Natural Science Foundation of Shanxi (201901D111440) and PhD Start‐Up Fund of Shanxi Provincial People′s Hospital (2018).

## Disclosure

All authors read and approved the final manuscript.

## Ethics Statement

The authors have nothing to report.

## Consent

The authors have nothing to report.

## Conflicts of Interest

The authors declare no conflicts of interest.

## Supporting information


**Supporting Information** Additional supporting information can be found online in the Supporting Information section. Table S1: Publication and citation profiles of leading countries. Table S2: Country clusters. Table S3. Bibliometric indicators of high‐impact journals. Table S4: Publication and citation profiles of high‐impact authors. Figure S1: Distribution of high‐frequency keywords across country collaboration clusters.

## Data Availability

All data generated or analyzed during this study are included in this article and Supporting Information files.
