# Supplementary material for: Visualization and Cluster Analysis of Peroxisome Proliferator–Activated Receptors in Colorectal Cancer: Research Trends and Future Directions
Source: Hum Mutat. 2026 Apr 24;2026:9971217. doi: 10.1155/humu/9971217 (PMC13107118; doi:10.1155/humu/9971217)
Supplement: Supplementary file 1 — Supporting Information Additional supporting information can be found online in the Supporting Information section. Table S1: Publication and citation profiles of leading countries. Table S2: Country clusters. Table S3. Bibliometric indicators of high‐impact journals. Table S4: Publication and citation profiles of high‐impact authors. Figure S1: Distribution of high‐frequency keywords across country collaboration clusters. [file HUMU-2026-9971217-s001.docx]

**Table S1 Publication and Citation Profiles of Leading Countries**

| **Country** | **Articles** | **Freq** | **SCP** | **MCP** | **MCP_Ratio** | **TP** | **TP_rank** | **TC** | **TC_rank** | **Average Citations** |
| --- | --- | --- | --- | --- | --- | --- | --- | --- | --- | --- |
| USA | 386 | 0.28 | 306 | 80 | 0.207 | 1347 | 1 | 28461 | 1 | 73.7 |
| CHINA | 306 | 0.222 | 271 | 35 | 0.114 | 1060 | 2 | 7323 | 3 | 23.9 |
| JAPAN | 161 | 0.117 | 146 | 15 | 0.093 | 456 | 3 | 8181 | 2 | 50.8 |
| ITALY | 81 | 0.059 | 66 | 15 | 0.185 | 293 | 4 | 2865 | 4 | 35.4 |
| KOREA | 60 | 0.043 | 48 | 12 | 0.2 | 200 | 6 | 1600 | 8 | 26.7 |
| GERMANY | 54 | 0.039 | 36 | 18 | 0.333 | 201 | 5 | 2655 | 6 | 49.2 |
| FRANCE | 38 | 0.028 | 28 | 10 | 0.263 | 141 | 7 | 2777 | 5 | 73.1 |
| INDIA | 30 | 0.022 | 25 | 5 | 0.167 | 77 | 8 | 618 | 15 | 20.6 |
| UNITED KINGDOM | 22 | 0.016 | 13 | 9 | 0.409 | 64 | 11 | 2161 | 7 | 98.2 |
| CANADA | 18 | 0.013 | 17 | 1 | 0.056 | 63 | 12 | 1118 | 10 | 62.1 |
| NETHERLANDS | 18 | 0.013 | 12 | 6 | 0.333 | 76 | 9 | 773 | 13 | 42.9 |
| SPAIN | 18 | 0.013 | 17 | 1 | 0.056 | 75 | 10 | 819 | 12 | 45.5 |
| POLAND | 16 | 0.012 | 11 | 5 | 0.313 | 41 | 18 | 363 | 19 | 22.7 |
| SWITZERLAND | 15 | 0.011 | 11 | 4 | 0.267 | 50 | 16 | 964 | 11 | 64.3 |
| AUSTRALIA | 12 | 0.009 | 10 | 2 | 0.167 | 39 | 19 | 626 | 14 | 52.2 |
| BRAZIL | 12 | 0.009 | 9 | 3 | 0.25 | 18 | 26 | 345 | 21 | 28.8 |
| IRAN | 12 | 0.009 | 11 | 1 | 0.083 | 61 | 13 | 167 | 27 | 13.9 |
| BELGIUM | 10 | 0.007 | 6 | 4 | 0.4 | 31 | 22 | 1272 | 9 | 127.2 |
| DENMARK | 10 | 0.007 | 9 | 1 | 0.1 | 57 | 14 | 350 | 20 | 35 |
| SWEDEN | 10 | 0.007 | 7 | 3 | 0.3 | 45 | 17 | 344 | 22 | 34.4 |

Note(s): Articles: Publications of Corresponding Authors only. Freq: Frequence of Total Publications. MCP_Ratio: Proportion of Multiple Country Publications. TP: Total Publications. TP_rank: Rank of Total Publications. TC: Total Citations. TC_rank: Rank of Total Citations. Average Citations: The average number of citations per publication.

**Supplementary Table S2.** Country Clusters

| **Country** | **Cluster** |
| --- | --- |
| Argentina | 10 |
| Australia | 4 |
| Austria | 6 |
| Belgium | 1 |
| Brazil | 1 |
| Canada | 7 |
| China | 8 |
| Colombia | 1 |
| Croatia | 1 |
| Cuba | 1 |
| Czech republic | 4 |
| Denmark | 2 |
| Egypt | 5 |
| Finland | 6 |
| France | 9 |
| Germany | 2 |
| Greece | 1 |
| Hungary | 1 |
| India | 7 |
| Iran | 7 |
| Ireland | 8 |
| Israel | 2 |
| Italy | 2 |
| Japan | 5 |
| Kuwait | 5 |
| Malaysia | 3 |
| Mexico | 10 |
| Monaco | 9 |
| Montenegro | 9 |
| Netherlands | 6 |
| New Zealand | 8 |
| North Ireland | 4 |
| Norway | 2 |
| Poland | 1 |
| Portugal | 1 |
| Russia | 2 |
| Saudi Arabia | 4 |
| Scotland | 3 |
| Singapore | 3 |
| Slovakia | 4 |
| South Africa | 5 |
| South Korea | 4 |
| Spain | 2 |
| Sweden | 3 |
| Switzerland | 3 |
| Thailand | 5 |
| Turkey | 6 |
| United Arab Emirates | 7 |
| UK | 3 |
| Ukraine | 1 |
| USA | 10 |
| Wales | 3 |

Note(s): Countries with different cluster number belong to different clusters.

**Table S3 Bibliometric Indicators of High-Impact Journals**

| **Journal** | **H_index** | **IF 2023** | **JCR 2023** | **TP** | **TP_rank** | **TC** | **TC_rank** | **PY_start** |
| --- | --- | --- | --- | --- | --- | --- | --- | --- |
| CANCER RESEARCH | 39 | 12.5 | 1 | 45 | 1 | 2973 | 2 | 1999 |
| JOURNAL OF BIOLOGICAL CHEMISTRY | 27 | 4 | 2 | 33 | 2 | 3165 | 1 | 2000 |
| CARCINOGENESIS | 25 | 3.3 | 2 | 29 | 3 | 1045 | 8 | 1998 |
| CLINICAL CANCER RESEARCH | 22 | 10 | 1 | 24 | 6 | 677 | 13 | 2000 |
| INTERNATIONAL JOURNAL OF CANCER | 20 | 5.7 | 1 | 25 | 5 | 621 | 15 | 2001 |
| PLOS ONE | 20 | 2.9 | 1 | 27 | 4 | 585 | 16 | 2011 |
| ONCOGENE | 18 | 6.9 | 1 | 21 | 8 | 867 | 10 | 2003 |
| INTERNATIONAL JOURNAL OF ONCOLOGY | 17 | 4.5 | 1 | 24 | 7 | 338 | 28 | 2000 |
| MOLECULAR CANCER THERAPEUTICS | 17 | 5.3 | 1 | 18 | 9 | 313 | 31 | 2006 |
| PROCEEDINGS OF THE NATIONAL ACADEMY OF SCIENCES OF THE UNITED STATES OF AMERICA | 16 | 9.4 | 1 | 16 | 14 | 2390 | 3 | 2000 |
| BIOCHEMICAL AND BIOPHYSICAL RESEARCH COMMUNICATIONS | 13 | 2.5 | 3 | 16 | 12 | 921 | 9 | 2000 |
| ANTICANCER RESEARCH | 12 | 1.6 | 4 | 17 | 10 | 252 | 41 | 2002 |
| BMC CANCER | 12 | 3.4 | 2 | 14 | 16 | 195 | 65 | 2003 |
| GASTROENTEROLOGY | 12 | 25.7 | 1 | 13 | 21 | 1231 | 6 | 1998 |
| MOLECULAR PHARMACOLOGY | 12 | 3.2 | 2 | 14 | 17 | 269 | 36 | 2002 |
| WORLD JOURNAL OF GASTROENTEROLOGY | 12 | 4.3 | 1 | 16 | 15 | 329 | 29 | 2003 |
| BRITISH JOURNAL OF CANCER | 11 | 6.4 | 1 | 12 | 22 | 454 | 22 | 2000 |
| ONCOTARGET | 11 | NA | NA | 14 | 18 | 256 | 40 | 2015 |
| BIOCHEMICAL PHARMACOLOGY | 10 | 5.3 | 1 | 11 | 26 | 296 | 33 | 2005 |
| CANCER LETTERS | 10 | 9.1 | 1 | 12 | 23 | 437 | 24 | 2004 |

Note(s): H_index: The h-index of the journal, which measures both the productivity and citation impact of the publications. IF: Impact Factor, indicating the average number of citations to recent articles published in the journal. JCR_Quartile: The quartile ranking of the journal in the Journal Citation Reports, indicating the journal's ranking relative to others in the same field (Q1: top 25%, Q2: 25%-50%, Q3: 50%-75%, Q4: bottom 25%). TP: Total Publications. TP_rank: Rank of Total Publications. TC: Total Citations. TC_rank: Rank of Total Citations. Average Citations: The average number of citations per publication. PY_start: Publication Year Start, indicating the year the journal started publication.

**Table S4 Publication and Citation Profiles of High-Impact Authors**

| **Author** | **h_index** | **g-index** | **m-index** | **PY_start** | **TP** | **TP_Frac** | **TP_rank** | **TC** | **TC_rank** |
| --- | --- | --- | --- | --- | --- | --- | --- | --- | --- |
| GONZALEZ FRANK J. | 16 | 16 | 0.889 | 2007 | 16 | 1.81 | 1 | 937 | 7 |
| PETERS JEFFREY M. | 16 | 16 | 0.889 | 2007 | 16 | 1.87 | 1 | 799 | 10 |
| DUBOIS RN | 14 | 15 | 0.519 | 1998 | 15 | 3.58 | 3 | 2235 | 3 |
| COLANTUONI VITTORIO | 10 | 10 | 0.625 | 2009 | 10 | 1.1 | 6 | 387 | 16 |
| ELING TE | 10 | 11 | 0.385 | 1999 | 11 | 2.36 | 5 | 819 | 9 |
| FISCHER SM | 9 | 9 | 0.36 | 2000 | 9 | 1.35 | 8 | 871 | 8 |
| SARRAF P | 9 | 9 | 0.333 | 1998 | 9 | 1.06 | 8 | 2816 | 1 |
| BAEK SEUNG JOON | 8 | 10 | 0.421 | 2006 | 10 | 2 | 6 | 183 | 20 |
| GUPTA RA | 8 | 8 | 0.296 | 1998 | 8 | 1.33 | 11 | 1451 | 4 |
| SABATINO LINA | 8 | 9 | 0.5 | 2009 | 9 | 1.01 | 8 | 334 | 17 |
| SAFE STEPHEN | 8 | 8 | 0.421 | 2006 | 8 | 1.61 | 11 | 309 | 18 |
| BAEK SJ | 7 | 7 | 0.292 | 2001 | 7 | 1.44 | 15 | 648 | 11 |
| BASSAGANYA-RIERA JOSEP | 7 | 8 | 0.368 | 2006 | 8 | 1.88 | 11 | 598 | 12 |
| HONTECILLAS RAQUEL | 7 | 8 | 0.368 | 2006 | 8 | 1.88 | 11 | 598 | 12 |
| HOU YONGZHONG | 7 | 12 | 0.538 | 2012 | 12 | 1.97 | 4 | 390 | 15 |
| KOHNO H | 7 | 7 | 0.292 | 2001 | 7 | 1.16 | 15 | 1093 | 5 |
| MUELLER E | 7 | 7 | 0.259 | 1998 | 7 | 0.76 | 15 | 2547 | 2 |
| TANAKA T | 7 | 7 | 0.292 | 2001 | 7 | 1.16 | 15 | 1093 | 5 |
| TANAKA TAKUJI | 7 | 7 | 0.368 | 2006 | 7 | 1.13 | 15 | 229 | 19 |
| WANG DINGZHI | 7 | 7 | 0.368 | 2006 | 7 | 1.67 | 15 | 451 | 14 |

Note(s): H_index: The h-index of the journal, which measures both the productivity and citation impact of the publications. g_index: The g-index of the journal, which gives more weight to highly-cited articles. m_index: The m-index of the journal, which is the h-index divided by the number of years since the first published paper. TP: Total Publications. TP_rank: Rank of Total Publications. TC: Total Citations. TC_rank: Rank of Total Citations. Average Citations: The average number of citations per publication. PY_start: Publication Year Start, indicating the year the journal started publication.


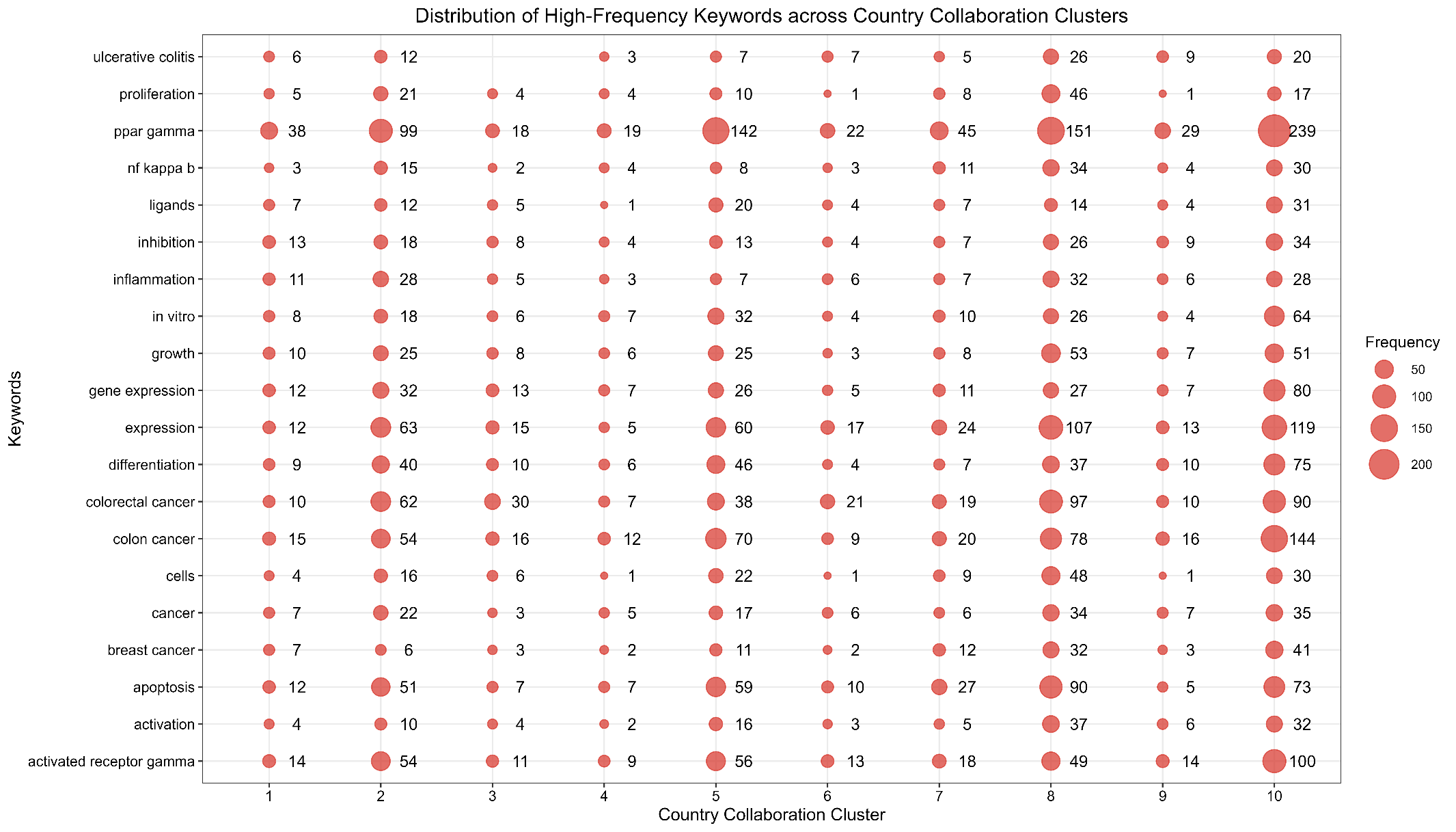


**Figure S1.** Distribution of High-Frequency Keywords across Country Collaboration Clusters. The horizontal axis represents the country-cooperative clusters (10 clusters in total), and the vertical axis represents the high-frequency keywords. The size of the circle reflects the frequency of the keyword in the corresponding country-cooperative cluster; the larger the circle, the higher the frequency. The number next to the circle indicates the specific number of times the keyword appears in the corresponding cluster.
